# Comparison Study of Airway Reactivity Outcomes due to a Pharmacologic Challenge Test: Impulse Oscillometry versus Least Mean Squared Analysis Techniques

**DOI:** 10.1155/2013/618576

**Published:** 2013-04-11

**Authors:** Elena Rodriguez, Charrell M. Bullard, Milena H. Armani, Thomas L. Miller, Thomas H. Shaffer

**Affiliations:** ^1^Nemours Research Lung Center, Nemours/Alfred I. duPont Hospital for Children, Wilmington, DE 19803, USA; ^2^Nemours Biomedical Research, Nemours/Alfred I. duPont Hospital for Children, Wilmington, DE 19803, USA; ^3^Division of Clinical Pharmacology, Thomas Jefferson University, Philadelphia, PA 19107, USA; ^4^Division of Neonatology, Thomas Jefferson University Hospital, Philadelphia, PA 19107, USA; ^5^Department of Pediatrics, Thomas Jefferson University, Philadelphia, PA 19107, USA

## Abstract

The technique of measuring transpulmonary pressure and respiratory airflow with manometry and pneumotachography using the least mean squared analysis (LMS) has been used broadly in both preclinical and clinical settings for the evaluation of neonatal respiratory function during tidal volume breathing for lung tissue and airway frictional mechanical properties measurements. Whereas the technique of measuring respiratory function using the impulse oscillation technique (IOS) involves the assessment of the relationship between pressure and flow using an impulse signal with a range of frequencies, requires less cooperation and provides more information on total respiratory system resistance (chest wall, lung tissue, and airways). The present study represents a preclinical animal study to determine whether these respiratory function techniques (LMS and IOS) are comparable in detecting changes in respiratory resistance derived from a direct pharmacological challenge.

## 1. Introduction

The use of animal models for studying respiratory mechanics under airway challenge tests has led to a sudden increase of information regarding the behavior of the different areas of the respiratory system. Despite the large amount of research in the adult and pediatric groups, we still lack significant knowledge in the neonatal subgroup. The present study represents a preclinical animal study to determine whether two respiratory function techniques are comparable in detecting changes in respiratory resistance derived from a direct pharmacological challenge.

The technique of measuring transpulmonary pressure and respiratory airflow with esophageal manometry, airway manometry, and pneumotachography has been previously described [1]. Transpulmonary pressure derived from proximal airway pressures and intrapulmonary esophageal pressure detected from a water-filled catheter [2, 3] are measured by differential pressure transducers. The airflow is measured with a low dead-space volume pneumotachometer and a differential pressure transducer. The least mean squared analysis (LMS) has been used broadly in both preclinical and clinical settings for the evaluation of neonatal lung function during tidal volume breathing [1, 4, 5].

The technique of measuring respiratory function using forced oscillation technique (FOT), or impulse oscillometry, involves the assessment of the relationship between pressure and flow using a forced/impulse signal composed with a range of frequencies. The response to this signal is called the respiratory impedance, which is the frequency-dependent relationship between transrespiratory pressure and flow. The impedance of the respiratory system includes the respiratory resistance (*R*_rs_) and the respiratory reactance. The *R*_rs_ is the component of the pressure-flow relationship of the pressure oscillation that is in phase with the airflow; it is measured by the forward sound impulse and includes the total resistance of the respiratory system (chest wall, lung tissue, and airways). The technique was introduced in the initial study conducted by Dubois et al. in the 1950s [6].

Since then, many advances have been made in refining and using this technique for clinical research. Further modification of the technique has included the introduction of a computer friendly device that applies a respiratory pressure impulse, the impulse oscillometry system (IOS), which differs from the initial FOT in its setup. It uses signals lasting 5 ms and containing 5 Hz harmonics up to 50 Hz [7].

Multiple authors have published data using these oscillatory techniques for studies that evaluated airway and respiratory tissue mechanic responses after airway challenges in adolescents and children [8–16]. The techniques differentiated between proximal and distal airway obstruction and are a sensitive approach to determine bronchial hyperreactivity. In this regard, it has been demonstrated that oscillation does not modify the airway smooth muscle tone [17].

Despite the variable use of reference values, the techniques hold promise because the patients do not need to complete respiratory maneuvers, measurements are taken during quiet respiration, and invasive procedures (e.g., placement of an esophageal balloon) are not required to measure the resistance of the respiratory tract. Despite the amount of past research to establish these techniques as valid methods for assessment of respiratory function in both children and infants [18–25], additional validation is required for the neonatal population.

Significant remodeling of the respiratory system occurs even after birth in humans [26] and animals [27]; however, when compared with adults, neonates/infants have an increased upper airway resistance, increased lower airway resistance, decreased lung volume, decreased efficiency of respiratory muscles, and increased chest wall compliance. Extrapolation of outcomes regarding respiratory physiology-function and pharmacological challenge is not feasible.

The neonatal subgroup has been the focus of recent work, and published data supports the investigation of the oscillometry assessment in the youngest patients, with some technical modifications, which included the stimulation of a minimum of 2-second breathing pause at the end of inspiration using the Hering-Breuer inflation reflex (reflex triggered to prevent overinflation of the lungs by creating an apneic pause) in unsedated neonates by means of a shutter valve and a face mask [28, 29]. The IOS approach in neonates has not been validated against an accepted “gold standard” technique of respiratory function measurement (i.e., the use of an esophageal balloon to determine transpulmonary pressures to separate the chest wall and lung components of the resistance spectrum).

Appropriately, the development and validation of oscillometry techniques for clinical use in neonates will allow assessment of respiratory function in this group of patients to accurately measure lung development, respiratory disease, and the response to drug or therapeutic challenges. The clinical applicability of the neonatal piglet model has been extensively studied for developmental respiratory biological processes and pre-clinical endpoints by our group [30] as well as numerous other investigators [31–37]. However, to validate this methodology for broad clinical use, we believe that accurate in vivo testing (a neonatal animal model) of an airway challenge is warranted.

Therefore, the main aims of this study were to detect significant pharmacological increases in resistance (% of change) with both respiratory function techniques and to assess the level of agreement between them in detecting this airway reactivity outcome. The overall study aim is to validate the IOS technique as a noninvasive means of evaluating respiratory mechanics in the neonatal setting through a range of resistance values representing the airway and tissue mechanical proprieties; this validation is to be compared with an older technique that is still currently accepted as a standard for respiratory mechanics evaluation but requires patient cooperation and placement of an esophageal pressure catheter [2].

To explore this gap in research, we conducted this pilot study to investigate the hypothesis that the IOS (new technique) could be interchangeably used to measure the effects of airway reactivity in neonatal settings.

## 2. Methods and Materials

### 2.1. Animal Preparation and Instrumentation Protocol

Neonatal piglets (*n* = 8, 6.6 ± 0.8 kg, and 20 ± 1 days of age) were anesthetized, intubated by tracheotomy, and allowed to breathe spontaneously. Piglets were anesthetized initially with two 1 mL/kg intramuscular injections, separated by 10 min, of an anesthesia cocktail (ketamine: 23 mg/kg; acepromazine: 0.58 mg/kg; xylazine: 0.8 mg/kg [KAX]) adapted from previously described piglet protocols [30, 38]. Local anesthesia was delivered to the skin and soft tissues around the surgical sites with 0.5% lidocaine HCl (4 mg/kg). For venous access and arterial blood sampling, respectively, 5- or 8-French umbilical catheters were inserted into the external jugular vein and carotid artery. A tracheotomy was performed to allow for continued spontaneous breathing through a 3.0- to 5.0-mm-ID endotracheal tube (ETT) (Hi-LoTM Jet tube; Mallinckrodt, Saint Louis, MO, USA). The size of the trachea in this model facilitated the use of a standard neonatal/pediatric ETT. The tracheotomy procedure was necessary to ensure a tight seal for more accurate measures of respiratory mechanics and to eliminate upper airway shunting and flow contribution.

**Figure 1:**
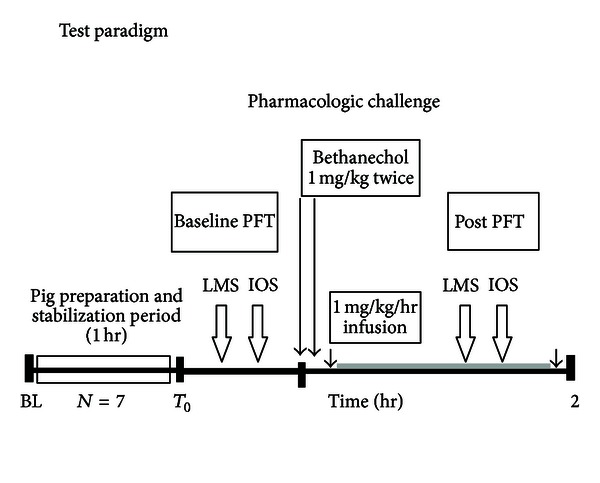
A schematic diagram illustrating the current test paradigm. LMS, least mean squares; IOS, impulse oscillatory system; PFT, pulmonary function testing; *N*, number of animals; BL, baseline; Post, after bethanechol administration. Time between the first and second bethanechol doses was 10 min.

Subsequent anesthesia was maintained with intravenous infusion of KAX at 0.4 mL/kg/hr. Maintenance fluid was provided by a continuous venous infusion of 5% dextrose solution at a rate of 6 mL/kg/hr. Arterial blood pressure was monitored by attaching the arterial catheter to a standard pressure transducer via bedside patient monitor (model M1175A, Hewlett Packard, Palo Alto, CA, USA). ECG electrodes were also placed for monitoring the heart rate and cardiac rhythm and to detect any significant bradycardia. Throughout the protocol, the animal's rectal temperature was monitored and maintained at 37-38°C on a radiant warmer bed (Resuscitaire; Hill-Rom Air-Shields, Hatboro, PA, USA). Once the physiologic stability of the animal was confirmed by the analysis of the arterial blood chemistry (Stat profile; Nova Biomedical, Waltham, MA, USA), ECG, and blood pressure monitoring; the animal's respiratory measurements were recorded as described below. Intravenous midazolam was used as an anxiolytic to ease the work of breathing. Following the completion of the protocol, animals were euthanized with pentobarbital (50 mg/kg) and saturated potassium chloride (2 mEq). All procedures for the animal preparation were approved by the Institutional Animal Care and Use Committee. Functional respiratory parameters of interest were recorded after the animal was stabilized. The piglet then was connected to the PEDS circuit for baseline measurements. Immediately following LMS baseline assessment (average of 5 min), the piglet was connected to the IOS system for baseline oscillatory measurements, and testing was performed as soon as the transrespiratory pressure returned to 10 cmH_2_O as described below. Also, Figure 1 illustrates the test paradigm that was followed in order to compare respiratory function assessment between the LMS and IOS methods both before and after pharmacologically induced airway constriction.

### 2.2. Respiratory Function Assessment-Least Mean Squared Analysis

For the conventional tidal breathing resistance measurements, the ETT was connected to the pneumotachometer port. A three-valve stopcock was used to block the pressure port. The negative flow port was manually occluded to prevent flow artifact since there was no additional flow via ventilator tubing. A water-filled balloon was placed via the mouth into the esophagus to measure the intrathoracic pressure during respiration. Optimum placement of the balloon was confirmed by real-time monitoring of pressure tracings, using criteria of maximum negative deflection during inspiration with minimum cardiac artifacts. Only spontaneous breaths (at least 10 breaths) were analyzed, and breaths with distortion of the signal were excluded from the average data.

Piglets were observed for 30 seconds on the circuit. Respiratory volumes were determinated by electronic integration of flow signals, and the following parameters were calculated: dynamic pulmonary resistance (*R*) in cmH_2_O/L/s, respiratory rate, tidal volume, and minute ventilation. Resistance values were recorded while positive pressure was applied to induce opposition to flow and calculated by least mean squared (LMS) algorithms incorporated into the computer system.

### 2.3. Respiratory Function Assessment-Impulse Oscillometry Analysis

The impedance of the total respiratory system was measured using a commercially available IOS that has been described previously. During tidal breathing through an ETT, an impulse generator delivered brief pulses at intervals of 0.2 sec, superimposed on the spontaneous breathing pattern. The digitalized pressure and flow signals were fed into the fast Fourier transformation, where 32 samples were considered. For experimental proposes, no system correction was used. Daily calibration using a calibration pump (3.0 ± 0.01 L SD, Jaeger; Höechberg, Germany) and a reference impedance of 10 cmH_2_O/L/s were performed, and a maximum error of 10% was permitted. Piglets were connected via ETT adapter to the pneumotach of the IOS, were tested for 30 seconds on the circuit with a transrespiratory pressure of 10 cmH_2_O, and were saved for later evaluation; epoch of measurements was discarded if the time flow and pressure pattern in the primary data time and time results trends showed interruption of the oscillatory signal, which occurred occasionally (approximately 10–15%) in the current study. In the evaluation phase, the minimum time period for reporting these measurements was 2 seconds. The impedance of the respiratory system (*Z*_rs_) and *R*_rs_ in cmH_2_O/L/s were calculated at 3, 5, 10, 15, 20, 25, and 35 Hz. Replicate oscillatory values were performed by JLAB 4.65.1.0 software (Erich Jaeger GmbH, Würzburg, Germany) and analyzed by JLAB 5.20.0.52 software (VIASYS Healthcare GmbH, Höechberg, Germany). Since parameters were assessed within the same piglet over time/delta changes, a correction for the impedance of the ETT was not needed.

Each piglet provided a matched set of data from the MasterScreen PFT IOS (Jaeger, Höechberg, Germany) and the PEDS pulmonary function unit (MAS, Hatfield, PA) for the oscillometry and LMS analyses, respectively. Each piglet was measured with each device under two conditions: baseline (pre) and after intervention (post).

Following baseline measures, bethanechol was used as the intervention to pharmacologically induce bronchoconstriction and subsequent elevated airway resistance. Bethanechol powder (carbamyl-*β*-methylcholine, Sigma-Aldrich) was mixed with saline (5 mg of bethanechol : 1 mL of saline) and administered as two intravenous injections at a dose of 1 mg/kg of bethanechol chloride in 0.2 mL/kg of saline set 10 min apart to achieve an increase in the respiratory resistance. Decay in the elevated resistance was compensated with an infusion of 1 mg/kg/hr of bethanechol chloride started through the venous line following the initial bolus. This dosing regimen was adapted from a previously described piglet protocol [30]. To compare the two methodologies described herein, we were interested in establishing a large range of pulmonary changes (baseline to maximum dose response without evoking significant cardiovascular changes) instead of a standard dose response assessment with small pulmonary changes.

Bethanechol bolus and maintenance dose were given as described above. Piglets were observed until stable bronchoconstriction was assumed (3–5 min after the second dose) when resistance increased at least 40% from baseline. Post-intervention LMS measurements were obtained, followed by IOS measurements, to prevent any unfavorable effect from the impulses on LMS parameters. Parameters of interest were documented for each technique, each time in the same sequence.

### 2.4. Statistics

Continuous variables were summarized overall by mean and standard error of the mean (SEM), unless indicated otherwise. All assumptions were tested for normality; in the case of violation of any assumption, nonparametric tests were performed. The delta changes were compared using paired *t*-test for the dependent continuous normal variables or Wilcoxon signed rank test for the nonnormal dependent variables, testing for positive mean or median differences, respectively. Significance was set at alpha level of 0.05 (1-sided). A comparative analysis of agreement between the two methods was detailed for the pharmacological challenge using the Bland-Altman plot analysis; the bias, or the average of the differences, and the 95% limits of agreement (LOA) were reported for all frequencies along with their linear regression analysis (*r* ^2^, slope, and *P* values). Statistical analysis was done using a combination of statistical software packages (GraphPad Prism version 5.02 for Windows; GraphPad Software, San Diego, CA, USA, and SPSS version 19; IBM, Chicago, IL, USA).

## 3. Results

In this animal model, measurements of respiratory mechanics were possible, and detection of an increase in resistance was achieved with both systems after bethanechol administration in seven of the piglets. One animal died during the postbethanechol measurements (post) secondary to cardiac arrest. Following intravenous (IV) administration of bethanechol, a small but statistically significant decrease was seen in heart rate (mean ± SD, 151 ± 12 versus 133 ± 6, −12%, *P* = 0.03) and arterial pressure (mean ± SD, 79 ± 5 versus 67 ± 5, −15%, *P* = 0.002) for the remaining 7 piglets; although these changes were not physiologically relevant for hemodynamic instability, there were no significant changes in respiratory rate, tidal volume, and minute ventilation following bethanechol administration.

**Figure 2:**
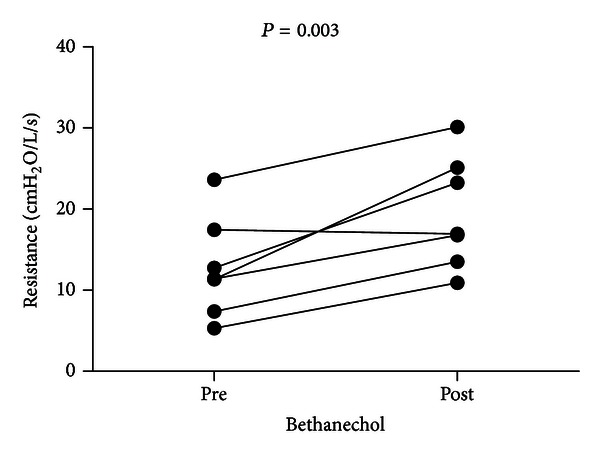
Plots of mean values for dynamic pulmonary resistance (resistance) before (pre) and after (post) bethanechol for each piglet. *P* = 0.003, paired *t*-test one tailed.

Respiratory mechanics measurements showed a significant increase in dynamic pulmonary resistance measured by the LMS (+53%, *P* = 0.003) following bethanechol administration (Figure 2). Pressure-volume loops also verified that, when compared with baseline, bethanechol administration resulted in increased pressure requirements as well as a widening and decrease in the slope of the pressure-volume loops. 

**Figure 3:**
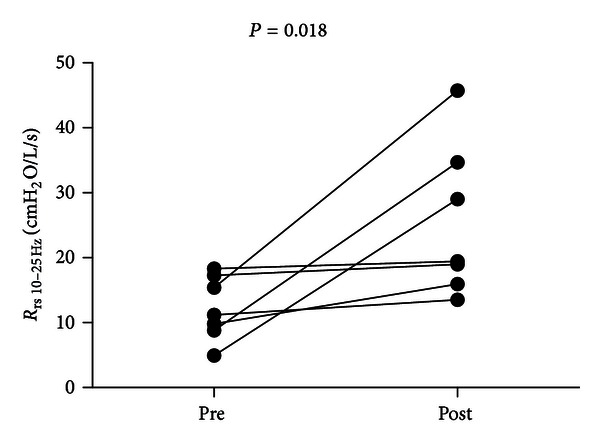
Plots of mean values for respiratory resistance (*R*_rs_) from 10 Hz to 25 Hz before (pre) and after (post) bethanechol for each piglet. *P* = 0.018, paired *t*-test one tailed for the average.

**Figure 4:**
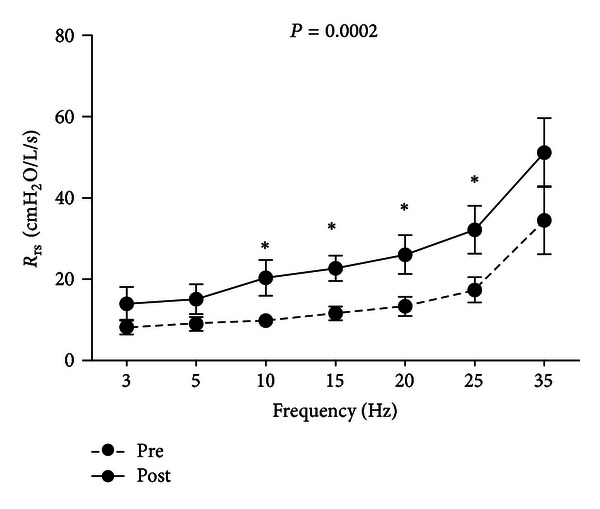
Plots of mean (±SEM) values for respiratory resistance (*R*_rs_), as a function of oscillatory frequency, before (pre) and after (post) bethanechol (*n* = 7). As shown, significant differences in the middle oscillatory frequencies range are displayed (*P < 0.05); paired *t*-test one tailed at each frequency and overall (*P* = 0.0002).

Following bethanechol administration, a significant overall increase in the respiratory resistance measured by IOS was demonstrated by positive changes in *R*_rs_ from 10 to 25 Hz (+96%, *P* = 0.018; Figure 3). Significant increases in specific respiratory resistances were demonstrated by positive changes in the intermediate frequency spectrum (frequencies: *R*_rs 10 Hz_ [+106%, *P* = 0.031], *R*_rs 15 Hz_ [+96%, *P* = 0.013], *R*_rs 20 Hz_ [+95%, *P* = 0.026], and *R*_rs 25 Hz_ [+85%, *P* = 0.037]). No statistically significant differences between the prebethanechol (pre) and postbethanechol (post) outcomes for any remaining frequencies were observed: *R*_rs 3 Hz_ (+67%, *P* = 0.131), *R*_rs 5 Hz_ (+67%, *P* = 0.09), and *R*_rs 35 Hz_ (+95%, *P* = 0.084), and the resistance spectrum of *R*_rs_ demonstrated increased positive frequency dependence (Figure 4).

**Figure 5:**
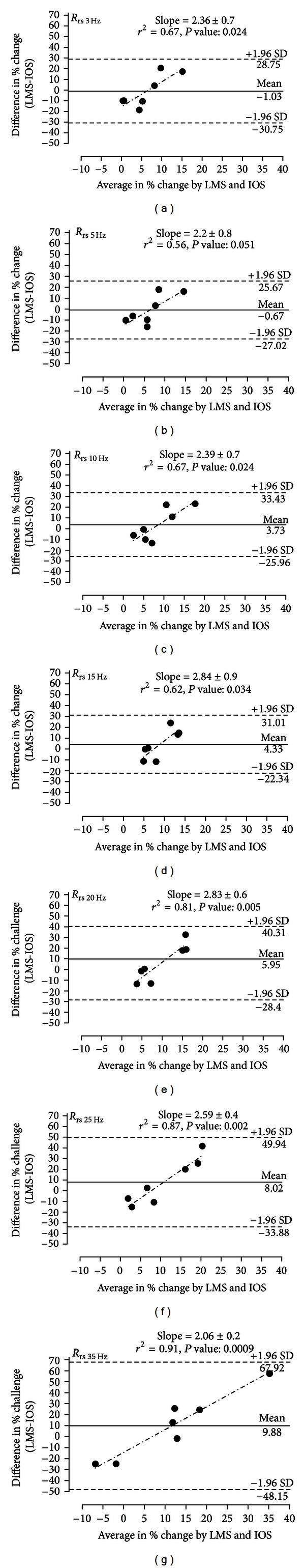
Bland-Altman plots constructed from the least mean square (LMS) and impulse oscillometry (IOS) data at each specific frequency (percentage of change) with mean (bias), ±LOA, and best-fit values of linear regression (slope, *r* ^2^, *P* value) after administration of intravenous bethanechol. LOA, limit of agreement; Upper LOA, mean difference (bias) + 1.96 SD; Lower LOA, mean difference (bias) −1.96 SD; *r* ^2^, *R* squares. The *P* value is testing the null hypothesis that the overall slope is zero.

The Bland-Altman analysis demonstrated that 95% of the differences between methods (IOS and LMS) lay within ±1.96 SD from the mean difference indicated by the LOA. Overall, there was a consistent linear sloped trend with a systematic bias (negative bias presented at lower values and positive bias presented at higher values of the measurement range) across the Bland-Altman plots, but within the LOA (Figure 5); this effect becomes less biased towards lower frequencies (Figures 5(a) and 5(b)) as do the slopes of the linear regression analysis.

## 4. Discussion

In the present study, we demonstrated that both respiratory function techniques assessed the effects of intravenous bethanechol on the respiratory system. Our results showed that delta changes of oscillometric resistances in the middle frequency range (*R*_rs 10_, 106%; *R*_rs 15_, 96%; *R*_rs 20_, 95%; *R*_rs 25_, 85%) were higher than LMS dynamic pulmonary resistance (*R*, 53%).

The smooth muscle of the respiratory tract has both parasympathetic and sympathetic innervations; carbamyl-*β*-methylcholine (bethanechol) is a choline ester and an agonist of muscarinic receptors that stimulates these receptors on the smooth muscle of the respiratory tract, causing airway narrowing and perhaps tissue constriction. Bethanechol belongs to the same group of drugs as acetylcholine, carbachol, and methacholine [39]. The piglet model used in this study was chosen based on its comparable size to the neonate/pediatric human and its common use for the investigation of ventilatory therapies [31–37].

In addition, bethanechol-induced airway challenges have been previously reported as an appropriate experimental intervention in the laboratory [40–44], and similar analogs (i.e., methacholine) have been used for clinical research purposes [45–47]. In humans, bethanechol is approved only for oral administration use; in veterinary medicine, it is available as an injection for subcutaneous (SC), intramuscular, or IV administration. Cardiac arrest is a severe, rare adverse reaction documented in humans, and in animals, it is a likely severe cholinergic reaction if given IV or in overdosage. It is recommended that atropine be immediately available when given IV or SC [48–51].

After bethanechol administration, a greater percentage of change was seen in airway resistance mainly represented by *R*_rs 10_–*R*_rs 25_ (+106–85%) when compared with the lung resistance mostly represented by *R*_rs 3_–*R*_rs 5_ (+69–63%), indicating a greater nonperipheral airway constriction response than peripheral airway/lung tissue response. Regarding the lung tissue contribution versus airway contributions to the total respiratory resistance, differences in the degree of tissue versus airway contribution exist between species [52, 53], including humans [54], and within induced-physiological and pharmacological conditions [44]; thus, caution should be taken in translating these findings between species.

It is widely recognized that the use of an esophageal balloon for resistance measurements separates the lung and chest wall component, therefore, excluding the chest wall resistance [4], while *R*_rs_ includes the resistance of the chest wall (i.e., lung resistance) and airway resistance. The lower the specific oscillatory frequency is, the more sensitive the measurement is to the periphery of the respiratory system. Measurement of the resistance using methods that require placement of an esophageal balloon for respiratory function testing procedures to assess the respiratory system in neonates is not easy. Noninvasive respiratory function techniques (i.e., impulse oscillometry) require less patient cooperation and provide more information about the total respiratory system resistance [19].

We also demonstrated that no pause in breathing was necessary for recording reliable data at low frequencies for *R*_rs_ using the impulse oscillation technique in neonatal piglets spontaneously breathing through an ETT. In this regard, the Hering-Breuer inflation reflex and face mask were not used in our study in comparison to previously citied studies in unsedated neonates/infants [28, 29].

The Bland-Altman analysis showed good agreement between the two techniques (IOS and LMS) for measuring resistance properties, and the agreement between these techniques depended on the oscillation frequency. From a mechanistic view point, *R* measured by the LMS in the study represents resistance of the lung tissue and airway frictional mechanical properties, whereas *R*_rs_ measured by IOS represents total respiratory resistance, so the two techniques are not directly interchangeable at lower frequencies. However, as mentioned in Section 3, the bias and slope decrease towards lower frequencies. In this regard, respiratory resistance at 5 Hz (*R*_rs 5_) and respiratory resistance at 3 Hz (*R*_rs 3_) may be closely interchangeable in the appropriate settings. However, the difference of ±1.96 SD from the mean difference for *R*_rs 35_ (Figure 5(g)) was demonstrated to be unacceptable for interchangeability.

Like many preliminary studies, the present study has some experimental limitations. Along these lines, the current study is an experimental preclinical model comparing the IOS and the LMS approach as an indicator of airway reactivity behavior to a pharmacologic intervention in a small number of animals. Additional work is required in the performance, reproducibility, and safety of the oscillation technique, including validation and qualification as a biomarker, before it can be proposed for standard clinical practice in the neonatal population.

## 5. Conclusions

In conclusion, we documented significant airway reactivity using both respiratory function techniques and demonstrated the magnitude of the change in airway resistance component at baseline and after administration of intravenous bethanechol. In a similar scenario, the measurements of respiratory resistance from the impulse oscillation technique (from 3 Hz to 25 Hz) may be used in lieu of the dynamic pulmonary resistance measurements from the LMS technique to determine resistance changes secondary to induced airway reactivity during spontaneous breaths.

The IOS technique as a noninvasive means of evaluating the different components of the respiratory system and pulmonary mechanics in the neonatal setting is very appealing; it gives further information through a range of resistance values representing the airway, tissue, and chest mechanical proprieties. However, further modification in the technique setup, incorporation of the appropriate references values for the neonatal population, and collaboration with experienced physicians and scientists in respiratory physiology/respiratory function techniques are required before broader clinical use is pursued in the neonatal population.
